# Progress in Medicine

**Published:** 1897-10-09

**Authors:** 


### Progress in Medicine.

### RHEUMATISM AND GOUT.

Stephen Mackenzie[1] takes a very wide view of rheumatism, which, he considers, appears under various forms at different periods of life, and in different persons, so that many other manifestations have just as much right to be true rheumatism as the type we find in some adults, and name rheumatic fever. Rheumatism should be, he says, a clinical term covering certain phenomena which occur in various combinations, and which are due to a common cause. Thus he includes under the term acute, sub-acute, and chronic rheumatism, various serous inflammations, chorea, or rheumatism of the brain, certain erythemas and purpura, which are the rheumatism of the skin, tonsillitis, or that of the throat, subcutaneous nodules, and possibly pneumonia. There are also, he holds, some other more rare manifestations, such as orchitis, acute thyroiditis, and some forms of nephritis. Several of these affections may occur together without the classical symptoms of rheumatism, but their frequent association with it, and their preceding or subsequent history, show their rheumatic origin. Apparently the author takes the incidence of classical rheumatism on the whole population as about 15 per cent., and argues that in these affections there is a history of rheumatism far exceeding this figure, and that this relation would be much more marked but for the fact that rheumatism frequently occurs unnoticed. Still, he confesses that cases, as for example of endocarditis, do arise where no rheumatic poison is likely. Thus, he mentions an endocarditis which arose from extension of the suppurative inflammation set up after opening a hydatid cyst. The great majority of cases, however, may be distinctly traced to an attack of rheumatism, or are followed by later and more defined ones.

Another view is taken by G. Parker,[2] who argues that, as we have been able to exclude from the rheumatic class gout and the septic joint diseases, so a fuller knowledge of these allied disorders shows many of them have definite characters and histories peculiar to them alone, and often sequelæ, such as nephritis and neuritis, unknown in rheumatism. The phrase, "a rheumatic history," is often misleading, as the history may have referred to some septic condition. He seeks to differentiate them by tracing out the characters of these diseases themselves, for rheumatism is at present undefinable. Henoch's purpura is taken as an instance of a type of disease often mistaken for rheumatism. There are violent gastric pains, diarrhœa, vomiting, pain and swelling around the joints, purpura, and hæmorrhages. But though many cases closely resemble rheumatism, we find nephritis and occasionally an enlarged spleen. Angio-neurotic œdema and rheumatoid arthritis are similarly very distinct from rheumatism, though superficially like it. They both present at times gastric crises, and show, like erythema multiforme and purpura, a preference for the female sex, which is not observed in rheumatism. The joint affections of septic type, such as gonorrhœal, variolous, and erysipelatous ones, are marked by very different histories from those of rheumatic origin, and the author quotes R. W. Marsden as showing that most of those following scarlatina are not rheumatic in symptoms, or sequelæ, if carefully examined. The erythematous affections, which Mackenzie considers to be rheumatic, often follow poisonous food, occur chiefly in women, and have special sequelæ of their own. Even on Mackenzie's own figures there are fifty per cent. which show no rheumatic histories or symptoms whatever. In the purpuras the hæmorrhagic form is associated with a bacterium, which Letzerich cultivated and, indeed, inoculated himself with it accidentally. The minor forms of purpura sometimes co-exist with rheumatism, but as they have different sequelæ, and are unaffected by salicylates, it is doubtful how far they are connected with it. A good example of the sort of disease commonly classed as rheumatic is seen in malignant endocarditis, the bacterial origin of which is now well recognised, though all the symptoms of classical rheumatism may be seen in it at certain stages. Recent work has shown that even simple endocarditis is produced in all kinds of infectious diseases, such as gonorrhœa, phthisis, and typhoid, and it is therefore of less value as evidence of rheumatism. Subcutaneous nodules have been found in syphilis and other diseases, and are not therefore pathognomonic of rheumatism. M. E. Paul[3] gives a case of a man with no history of rheumatism, and in perfect health except for a mitral bruit, who developed such large and numerous nodules that they gave inconvenience, and several were removed by a surgeon to relieve the discomfort.

**Treatment.**—A good deal has been written of late as to the possibility of replacing the salicylates by some remedy less irritating to the stomach and kidneys than the salicylates are. Hare[4] remarks that the oil of gaultheria, which contains 90 per cent. of salicylate of methyl, will act as a good substitute, but when taken by the mouth it is just as irritating. On the other hand, it is easily absorbed through the skin or by inhalation, and good results have been obtained not only in acute rheumatism but in gonorrhœal cases. He quotes the trials of it made by Linossier, who placed one or two drachms on lint next the skin, and covered it closely by a sheet of guttapercha and a bandage to ensure the absorption of the remedy. In this way relief of the symptoms followed much the same as when it was given by the mouth. In half an hour a reaction was found in the urine, and complete elimination took place in twenty-four hours, singing in the ears being noticed when the dose was large. Laserre,[5] to avoid the uncertainty of the quality of the oil of wintergreen, employs pure methyl salicylate made synthetically, and gives it by the mouth in doses of half a gramme in twenty-four hours, with spirit and syrup as a draught. This preparation is said to be free from the unpleasant odour of the oil of gaultheria, agreeable to take, and not irritating to the stomach. In peliosis rheumatica, Mühlbauer[6] finds that cases which resist ordinary remedies quickly yield to salipyrin, one of the new synthetic compounds. He takes the view that the disease is a distinct entity, and neither rheumatism or purpura.

**Rheumatoid Arthritis.**—For the arrest of this disorder Philpotts[7] has employed methylene blue, in doses of two grains twice daily after food, with great and rapid improvement of the condition. The cases were very obstinate ones, but under this treatment the disease seemed to be arrested, and the patients gained in weight. The method is of importance, not only from the difficulty of alleviating the sufferings of these patients, but with regard to the asserted bacterial origin of the disease. The methylene blue has already proved its value as an anti-microbic in malaria, cystitis, and other affections. Armstrong[8] reports the trial of it in several patients suffering from rheumatoid arthritis, and found it very successful in cases where there was reason to think that intestinal auto-intoxication existed, though less useful where the cause appeared to be uterine irritation, or post-influenzal conditions. At the German Congress of Internal Medicine, Baumler[9] discussed the pathology and treatment of the disease. He refers to the importance of atmospheric influences, to hereditary tendencies, and to its preference for the female sex, but he finds no direct connection existing with any known disease of the nervous system. The bacterial discoveries of Wohlman, Bannatyne, and others are interesting, but they do not as yet warrant us in asserting a single cause of the disease. Schüler reports an examination of the microbes present. He obtained similar results to those of Bannatyne, and succeeded in growing the bacilli and producing an arthritis by inoculation in rabbits, without, however, any change in the cartilages. Ott recommends an abundant diet, and aims at combatting the anæmia and strengthening the patient. Schüler finds that injections of guiacol into the joints cause the disappearance of large outgrowths, but the treatment is painful, and several injections are necessary. He adds as much as 15 c.c. of guiacol to iodoform emulsion for an injection into the knee, and repeats it, if required, six or eight times. In the smaller articulations a single injection will at times be sufficient.

**Purpura Hæmorrhagica.**—L. Weber[10] considers that the disease runs its course in from five to eight weeks unless a very large hæmorrhage occurs, with resulting collapse and œdema. This collapse will generally yield to the persevering use of saline injections and excitants such as camphor and caffeine. The disease is apt to come on in the course of convalescence from infectious disorders, and he thinks that it differs in degree only from purpura simplex. Still, it probably depends on some unknown intoxication, and the form which occurs with rheumatism is uninfluenced by salicylates. On the whole, the prognosis is good, and the most useful drug is the liquor ferri. G. Parker[11] mentions a case where a young man in good health developed an attack of this disease. Crops of dark-coloured bullæ and petechiæ came out for some weeks without interfering with the general health; but on their increasing he came into a hospital, and died next day from hæmorrhage in the brain through an extension of the purpura.

**Scurvy.**—The experience of Nansen's expedition has given rise to discussions on the causation of scurvy. Torup believed that it is due to ptomaines generated in badly-preserved foods, and great care was taken in selecting the preserved foods on which Nansen's party lived with complete success. A. E. Wright[12] shows that the herbivora have no protection against acid administered in their food, and quickly die if it is given artificially. In the carnivora there is a provision for neutralising acid by the waste ammonia of the body. In the human being this is less perfect, and if all vegetable food is cut off we should get an acid intoxication. Thus, he believes that scurvy is an acid intoxication. But Nansen has shown that fresh blood and raw flesh are an antidote; and Wright says that this is easily explicable, because blood is not only alkaline, but it produces salts akin to those obtained from lemon juice. However, as the explorers were often without fresh blood and flesh much of their time, the immunity may have been, after all, due to the carefully preserved foods which they consumed.

[1] Edin. and Med. Jour., p. 32, 1897. [2] Lancet, June 26. [3] Lancet, June 26. [4] Therap. Gaz., Feb. 14. [5] Med. Week, Feb. 25. [6] B. Med. Jour., March 13. [7] B. Med. Jour., March 27. [8] B. M. Jour., April 24. [9] Med. Week, June 18. [10] N. Y. Med. Jour., April 10. [11] Lancet, Jun 26. [12] Lancet, Feb. 27.

